# Novel Wet Micro-Contact Deprinting Method for Patterning Gold Nanoparticles on PEG-Hydrogels and Thereby Controlling Cell Adhesion

**DOI:** 10.3390/polym9050176

**Published:** 2017-05-15

**Authors:** Cigdem Yesildag, Christoph Bartsch, Gonzalo de Vicente, Marga C. Lensen

**Affiliations:** Technische Universität Berlin, Nanopatterned Biomaterials, Sekr. TC 1, Strasse des 17. Juni 124, 10623 Berlin, Germany; cigdem.yesildag@tu-berlin.de (C.Y.); Corynebacter@hotmail.de (C.B.); g.devicentelucas@tu-berlin.de (G.d.V.)

**Keywords:** gold nanoparticles, poly(ethylene glycol) hydrogel, micro-patterning, swelling, deprinting, cell adhesion

## Abstract

In the present work we introduce a novel method to create linear and rectangular micro-patterns of gold nanoparticles (Au NPs) on poly(ethylene glycol) (PEG) hydrogels. The strategy consists of removing Au NPs from defined regions of the silicon wafer by virtue of the swelling effect of the hydrogel. Using this method, which we denote as “Wet Micro-Contact Deprinting”, well-defined micro-patterns of Au NPs on silicon can be created. This resulting pattern is then transferred from the hard substrate to the soft surface of PEG-hydrogels. These unique micro- and nano-patterned hydrogels were cultured with mouse fibroblasts L929 cells. The cells selectively adhered on the Au NPs coated area and avoided the pure PEG material. These patterned, nanocomposite biointerfaces are not only useful for biological and biomedical applications, such as tissue engineering and diagnostics, but also, for biosensor applications taking advantage of surface plasmon resonance (SPR) or surface enhanced Raman scattering (SERS) effects, due to the optical properties of the Au NPs.

## 1. Introduction

Studying the interactions of micro-organisms, living cells or other biological systems, such as proteins or enzymes, with biomaterials is important for the design and development of novel biointerfaces. Depending on the surface characteristics, biomolecules exhibit specific or non-specific interactions at biointerfaces, where non-specific protein adsorption (NSPA) may lead to undesired phenomena (e.g., inflammation or biofouling) [1,2,3]. In order to prevent the non-specific biointeractions, we, among others, have exploited inert base materials, onto which micro- and nano-patterns are created that elicit specific recognition and selective cell binding to the biomaterial’s surface. The well-known and often used inert biomaterials, poly(ethylene glycol) (PEG)-based hydrogels, are particularly good candidates. PEG is highly resistant to non-specific protein adsorption, non-immunogenic, and non-toxic and is therefore widely used for drug delivery in medicine, in pharmacy, cell biological research and in the industry, mainly as care products [4,5].

PEG-based hydrogels (i.e., crosslinked PEG-networks that are insoluble in water, but have a great tendency to imbibe water) are attractive biomaterials to study cell behavior: adhesion, migration, and growth. Interestingly, although PEG-based materials are renowned for being non-adhesive to cells, we discovered that cells do in fact adhere to PEG-hydrogels when they are topographically or elastically patterned. For example, we observed that nano- and micro-topographies on the surfaces of PEG-hydrogels enabled adhesion of fibroblasts [6,7]. In addition, we have observed selective cell adhesion and migration on PEG-hydrogels with micro-patterns of elasticity fabricated by our Fill-Molding in Capillaries method (FIMIC) [8,9,10].

Besides topographic and elastic patterns, chemical modifications of PEG-based materials can also be exploited to control specific biointeraction. For instance, we have demonstrated that fibroblasts can be geometrically confined within anti-adhesive PEG-microstructures on adhesive polymeric surfaces [11]. Chen et al. reported that such a geometric confinement can be used to control cell fate [12], while Wang et al. later discovered that spacing and control of adhesive patches can even control cell differentiation [13]. The former scientists employed micro-contact printing (µ-CP), the versatile micro-patterning technique developed by Whitesides et al., a few decades ago [14]. Originally, µ-CP relies on the selective binding of thiol (–SH)-functionalized molecules to gold surfaces. The molecules were printed onto such gold surfaces by means of elastomeric stamps made out of poly(dimethyl)siloxane (PDMS) with a micrometer-sized relief.

Taking advantage of the selective binding of thiols to gold, Spatz et al. greatly miniaturized the chemical patterning of biointerfaces by fabricating periodic and aperiodic arrays of gold nanoparticles (Au NPs) as the template for biofunctionalization [15,16,17]. They revealed that integrin-mediated cell binding critically depends on the nanoscale availability of binding sites, which in their case were Au NPs that were functionalized with a cyclic RGD, the peptide sequence that is recognized by integrin receptors at the cell membrane. Notwithstanding, the impressive accuracy and the nanoscale of their study precision, the whole nanofabrication procedure involved several steps, sophisticated instruments and specially synthesized biomolecules and linkers [13,17,18].

We have therefore focused our efforts on the fabrication of designed patterns of Au NPs onto PEG-hydrogels without utilizing any kinds of linker molecules. In this contribution, we report on the successful creation of linear and rectangular micro-patterns of Au NPs on PEG-hydrogels and the application of these novel nanocomposite biomaterials in cell culture with fibroblasts. The great advantages of our strategy are based on its simplicity and versatility; in only a few easy steps, we can create well-defined patterns of Au NPs with predetermined sizes and shapes on PEG-gels that are tailor-made to possess the desired physico-chemical properties. The patterning methods are adapted from the most useful soft lithography techniques, as developed by us and others, using micro-structured elastomeric stamps, e.g., embossing, Micro-Molding in Capillaries (MIMIC) [19,20], and a variation on micro-contact deprinting (µ-CdP) [21]. It should be noted that the Au NPs, which are synthesized by the seeded growth method [22] and are stabilized by citrate, are used as such, without any specific or complicated biofunctionalization. Very recently, we discovered namely that fibroblasts readily adhere to such non-functionalized Au NPs on PEG-hydrogels [23].

The procedure to fabricate micro-patterns of Au NPs on PEG-hydrogels basically consists of two steps:

(1) Micro-patterning of Au NPs on a silicon wafer: For this, PEG-stripes on the silicon are adhered using an adaptation of the MIMIC method, which we called in a previous report: “adhesive embossing” [24]. Those stripes are subsequently removed by swelling of the hydrogel, a process that we coined as “wet micro-contact deprinting”, taking with them the Au NPs from the contact area and leaving a micrometer sized pattern of Au NPs-coated and non-coated stripes on the silicon wafer.
(2) Transfer of the micro-pattern of Au NPs from silicon onto a PEG-hydrogel: In this step, the prefabricated Au NPs-pattern on silicon is transferred as a whole to the surface of a PEG-hydrogel. The highest transfer efficiency is not achieved when a preformed PEG-hydrogel is employed, but rather when the PEG-hydrogel is formed from liquid PEG-precursors that are homogeneously spread on the micro-patterned silicon wafer, and then crosslinked by UV-curing (photoinitiated cross-linking). The crosslinked gel is separated from the hard substrate by the wet deprinting principle again, i.e., taking advantage of the hydrogel’s swelling effect.

## 2. Materials and Methods

### 2.1. Chemicals

Silicon wafers were purchased from Microchemicals GmbH (Ulm, Germany), Si-Masters were from Amo GmbH (Aachen, Germany), Trichloro(1H,1H,2H,2H-perfluorooctyl)silane, Tetrachloroaurate trihydrate (HAuCl_4_·3H_2_O), trisodium citrate (Na_3_C_6_H_5_O_7_), poly(ethylene glycol) diacrylate (PEGDA, Mw 575) and 2-hydroxy-4′-(2-hydroxyethoxy)-2-methylpropiophenone (photoinitiator-PI Irgacure 2959) were purchased from Sigma-Aldrich Chemie GmbH (Steinheim, Germany). (3-Aminopropyl)trimethoxysilane (APTMS) was bought from ABCR GmbH & Co. KG (Karlsruhe, Germany). Poly(dimethylsiloxane) (PDMS) and the curing agent are from Dow Corning GmbH (Wiesbaden, Germany). All the chemicals were used without further purification.

### 2.2. Seeded Growth Synthesis of Au NPs

#### 2.2.1. Synthesis of Au NPs Seeds

The citrate capped Au NPs seeds were synthesized as described by Bastús et al. [22]. Briefly, in a three-necked round bottom flask 0.17 mmol of trisodium citrate was dissolved in 75 mL of deionized water and heated for 15 min under vigorous stirring. A condenser was used to prevent the evaporation of the solvent. After boiling had commenced, 0.5 mL of a solution containing 0.63 mmol H[AuCl_4_]·3H_2_O solved in 25 mL of deionized water (precursor) was added to the trisodium citrate solution. The resulting pink mixture was kept stirring under reflux for an additional 10 min.

#### 2.2.2. Seeded Growth of Au NPs

Immediately after the synthesis of the Au NPs seeds was finished, the reaction was cooled down until the temperature of the solution reached 90 °C. Afterwards, 0.5 mL of the precursor solution was injected and the reaction was stirred for 30 min. This process was repeated twice. After the third addition of the precursor, the Au NPs solution was diluted by extracting 27.5 mL of the Au NPs solution and adding 26.5 mL of deionized water and 1 mL of a solution of 1.50 mmol trisodium citrate. This solution was then used as the seed for the subsequent growing step, repeating the whole process again. The reaction temperature was maintained at 90 °C during the growing steps. In that way, depending on the number of growing steps, spherical Au NPs with diameters up to 200 nm were possible to achieve. For the present study, we selected those with a diameter of 20 nm and 120 nm.

### 2.3. Coating of Silicon Wafers with Au NPs

The silicon wafers were silanized with an amino-silane layer via the vapor deposition method and then coated with Au NPs.

Before the silanization the silicon wafers were firstly washed with water, acetone and isopropanol and were then immersed into a mixture of H_2_O_2_:H_2_SO_4_ (3:7 *v*/*v*) for half an hour in order to activate the hydroxylate groups on the surface, washed first with water, then with isopropanol and dried under a stream of nitrogen.

The silicon wafers were silanized using the vapor method: The cleaned and activated wafers were placed in a clean petridish and then placed into a desiccator. Incidentally, in a small vial containing 1–2 drops of the silanizing agent APTMS was placed into the desiccator together with the silicon wafers. Subsequently, the desiccator was kept under vacuum for 2 h. After that, the silanized silicon wafers were washed with toluene and isopropanol and then dried under a flow of nitrogen.

On this self-assembled amino-silane layer, citrate-capped Au NPs were immobilized. For this, a droplet of Au NPs solution was spread on the amino-layer of the silicon wafer and left for approximately 1 h. After that the wafer was washed with water and isopropanol and dried under a flow of nitrogen. The citrate-capped Au NPs were bound to the amino-layer through electrostatic interactions.

### 2.4. Preparation of the PDMS Mold

**Figure 1:**
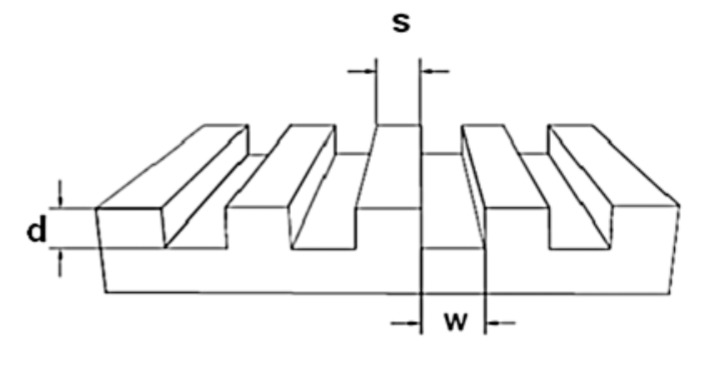
Structure of a silicon master.

For the preparation of the mold a micro relief-patterned master was necessary. The sizes of the masters are described with a three-numeric code: w-s-d = width of the grooves-spacing between the grooves-depth of the grooves, as shown in Figure 1. The surface of the master was silanized with trichloro(1H,1H,2H,2H-perfluorooctyl)silane in order to make the surface inert.

The PDMS mold was prepared by using a mixture of Sylgard 184 silicone elastomer and curing agent (10:1 *v*/*v*). In order to avoid bubbles the mixture was degassed in a desiccator, then casted on the silicon wafer and cured 2 h at 120 °C. The resulting PDMS mold was a negative copy of the silicon master.

### 2.5. Wet Micro-Contact Deprinting (Wet μ-CdP)

#### 2.5.1. MIMIC Approach

**Figure 2:**
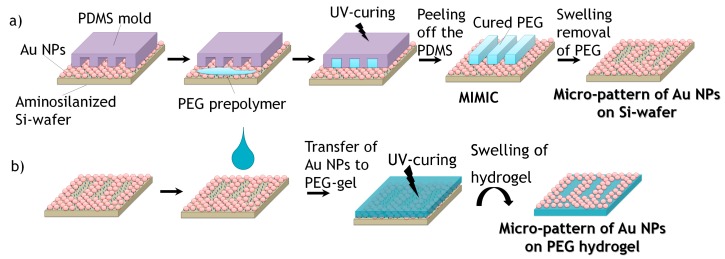
Schematic process of Wet Micro-Contact Deprinting (wet μ–CdP): (**a**) micro-patterning of gold nanoparticles (Au NPs) on silicon wafer via Micro-Molding in Capillaries (MIMIC) process; (**b**) transfer micro-pattern of Au NPs onto poly(ethylene glycol) (PEG)-hydrogel.

For creating defined micro-patterns of Au NPs on silicon wafers, micro-stripes of PEG-hydrogels were formed following the MIMIC process of Whitesides et al. [20]. For this, firstly, PDMS molds with a micro-pattern of lines were prepared by replication from silicon masters as described above. In order to attach microbars of PEG-hydrogel onto the surface of Au NPs-coated silicon substrates, first, a PDMS mold was placed gently on the surface with the micro-capillaries of the pattern facing it. A small droplet of PEGDA precursor mixed with 1 wt % photoinitiator Irgacure 2959 was placed at the entrance of the micro-channels of the PDMS-stamp in order to fill the empty channels. The filling of the micro-channels due to capillary forces was followed by UV-curing (365 nm, 6 W) in an oxygen-free atmosphere for 30 min. Afterwards, the PDMS mold was carefully peeled off from the silicon substrate revealing the cured inverse PEG-hydrogel stripes on the silicon surface. This first step of the process is shown schematically in Figure 2a.

#### 2.5.2. Swelling Removal of Hydrogels Micro-Lines in Water

The Au NPs-coated silicon wafers with micro-stripes of PEG-hydrogel were immersed in deionized water, in order to detach the embossed lines of PEG-hydrogel, together with the corresponding Au NPs that were in contact with the PEG-gels. Afterwards, the silicon wafers were rinsed with water and isopropanol followed by drying under a stream of nitrogen. In that way, line patterns of Au NPs on silicon wafers are achieved. This last step of the patterning process is shown in Figure 2a as well.

#### 2.5.3. Transference of Au NPs Pattern onto the Surface of PEG Hydrogel

A few drops of PEGDA precursor mixed with 1 wt % photoinitiator Irgacure 2959 were casted on the surface of the Au NPs patterned silicon wafer. Subsequently, the polymer was UV-cured as previously described. The created PEG-hydrogel on the silicon wafer was lifted off from the silicon surface by swelling of the hydrogel in water (Figure 2b).

**Figure 3:**
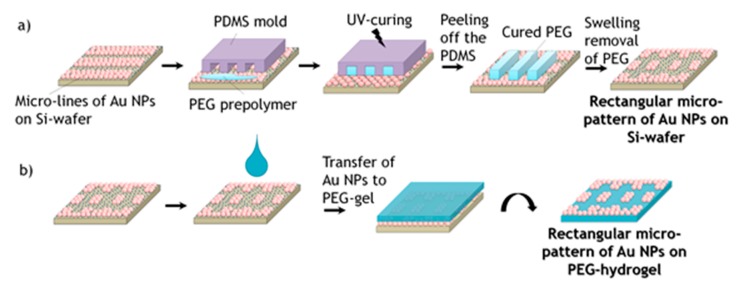
Schematic representation of the fabrication of rectangular patterns of Au NPs on PEG-hydrogels using wet μ-CdP process: (**a**) rectangular micro-patterning of Au NPs on silicon wafer via MIMIC process; (**b**) transfer of rectangular micro-pattern of Au NPs onto PEG-hydrogel.

In order to achieve square or rectangular structures, the wet μ-CdP procedure was done in the perpendicular direction with respect to the firstly created micro-lines (Figure 3), where the PEG hydrogel micro-stripes had crossed structures to the previous Au NPs lines. After the PEG prepolymer was cured, the PDMS mold was removed and the remaining PEG stripes were removed by swelling as described previously a square or rectangular micro-pattern of Au NPs on silicon wafer was achieved. The created square or rectangular micro-pattern of Au NPs was then again transferred from the silicon wafer onto the surface of PEG-hydrogel via swelling of the hydrogel in water (see Figure 3b).

### 2.6. Cell Culture

Mouse fibroblasts L929 (Dr. Lehmann, Fraunhofer Institute for Cell Therapy and Immunology, IZI, Leipzig, Germany) were cultured in RPMI 1640 medium with the addition of 10% Fetal Bovine Serum (FBS) and 1% Penicillin/Streptomycin (PS) in a cell culture plate (Carl Roth GmbH + Co. KG, Karlsruhe, Germany) in an incubator CB150 Series (Binder GmbH, Tuttlingen, Germany) at a controlled temperature (37 °C) and CO_2_ atmosphere (5%). Medium, sera and reagents were from PAA Laboratories GmbH, Germany, unless stated otherwise. Phosphate Buffered Saline solution (Dulbecco’s PBS) was purchased from Sigma-Aldrich Chemie GmbH (Steinheim, Germany). The cell counting chamber was from Marienfeld Superior (Paul Marienfeld GmbH & Co. KG, Lauda-Königshofen, Germany).

Once a confluency of 75% to 95% on a tissue culture plate was reached, the cell culture experiments were done. The cells were washed prior with PBS solution and treated for 2 min with trypsin-solution in the incubator, in order to detach the cells from the culture plate. The cell detachment was then controlled with optical microscopy. Then fresh cell medium was added into the cell trypsin mixture. This mixture was centrifuged for 4 min at 1300 rpm at 4 °C. Thereafter, the solvent was removed and the precipitation was dispersed in a new medium solution. In order to achieve a concentration of cells of 50,000 cells/mL, a cell counting chamber was used and the prepared biomaterials were cultured with these cells for 24 h.

#### Live/Dead Cyto-Toxicity Assay

The live/dead cytotoxicity assay is a fluorescence-based method for studying the viability of cells. Hereby the cells were stained with fluorescein diacetate (FBS) and propidium iodide (PI) molecules; a 1:1 *v*/*v* solution of these staining agents in PBS was prepared and added into the cell culture solution in a dark environment. Immediately after the mixtures were prepared, fluorescence images were taken. The FBS dissociates in the cytoplasma of the live cells into the green fluorescence molecules and due to the size and charge of the PI molecules they only can enter into the cell cytoplasm when the cell membrane is damaged and are bound to nucleic acids which appear then as red fluorescent color. In that way, the live cells appear as green-stained cells and dead cells as red-stained cells in a fluorescent microscope image.

### 2.7. Characterization Instruments

The samples containing Au NPs on silicon wafers were analyzed using a scanning electron microscope (SEM) (LEO982, Zeiss, Göttingen, Germany) and the optical parts of the microscope were from GEMINI Optics, USA. The measurements were performed using an Inlens detector operating at 15.0 kV. For the characterization of the surface topography of the micro-structured PEG hydrogels in swollen state, an atomic force microscope (AFM) (JPK instruments, Nanowizard II, Berlin, Germany) was used. The results of the cell culture experiments were monitored with an inverted optical microscope from Carl Zeiss, Germany and the analysis was done with the AxioVision V4.8.2 software (Carl Zeiss, Jena, Germany).

## 3. Results and Discussion

In this work, micrometer-sized, linear and rectangular patterns of Au NPs on PEG-hydrogels were created via the wet μ-CdP process, with the eventual goal to control specific adhesion of mouse fibroblast cells L929 on the created PEG-hydrogels. As mentioned in the introduction, the wet μ-CdP procedure consists of two main processes, namely (i) the MIMIC process to make the micro-patterns and (ii) the transfer of the resulting Au NPs patterns onto the surface of PEG-hydrogels. These two steps were schematically shown in Figure 2 for linear patterns and additionally in Figure 3 for rectangular patterns.

**Figure 4:**
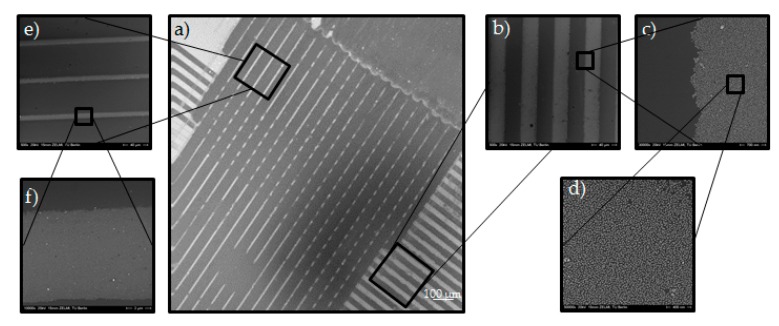
SEM images of a silicon wafer with micro-patterns of Au NPs (20 nm) using the wet μ-CdP procedure (size of the pattern of the poly(dimethyl)siloxane (PDMS) mold: 25 µm-25 µm-5 µm and 10 µm-20 µm-10 µm, resp.). Scale bars: (**a**) 100 µm; (**b**) 40 µm; (**c**) 700 nm; (**d**) 400 nm; (**e**) 40 µm; (**f**) 2 µm.

The first challenge was to create a well-ordered monolayer of Au NPs on silicon wafers. In this respect, a self-assembled monolayer of amino-terminated silanes (APTMS) was created via the vapor deposition method. A PDMS mold, which had micro-sized relief line-structures on the surface (as a replica of the silicon master, see Figure 1), was placed on the Au NPs coated silicon wafer. This mold had line-channels which were filled with the liquid PEG prepolymer through capillary forces and cured under UV-light. Afterwards, the PDMS mold was removed; leaving an ordered pattern of PEG micro-stripes on the Au NPs layered silicon wafer in respect to the MIMIC process. The key aspect for generating the desired Au NPs pattern relied on the wet deprinting of the adhered PEG micro-stripes which take off the Au NPs selectively on the area of contact, leaving a micro pattern of Au NPs in the untouched areas on the silicon wafer. Hereby, the cured PEG micro-stripes were removed by swelling in water. During the swelling, the Au NPs were taken away by the PEG-hydrogel micro-stripes and well-ordered Au NPs micro-lines or rectangles were achieved, which can be seen in the SEM images in Figure 4.

The PDMS mold that was used in Figure 4 had the sizes 25 µm-25 µm-5 µm (s-w-d) for filling the channels from the right to the left side and 10 µm-20 µm-10 µm for filling the channels from the top part to the bottom part (see Figure 4a). This pattern can be recognized in the SEM image in Figure 4a as a clear contrast between Au NPs lines and vacant areas. Figure 4e,d is enlarged views of 20 µm-10 µm pattern. In the center part of Figure 4a the rectangular Au NPs pattern with pattern sizes of 10 and 25 µm and distances of 20 and 25 µm is observable in accordance with the used silicon master. SEM analysis showed no Au NPs on the surface of the silicon wafer on the area where the PEG-stripes were placed which indicates to a complete transfer of the Au NPs. from silicon wafer to the hydrogel. It should be noted that the pattern is obviously not completely perfect; besides areas where the Au NPs were not removed where they should have been, and the rectangles were not successfully formed, there are some areas visible (dark grey in Figure 4a), where too many Au NPs have been removed in the deprinting process. This is most likely due to the formation of a small scum layer; the PEG-precursors must have creeped out of the channels on the PDMS mold and wetted the area between channels [25].

**Figure 5:**
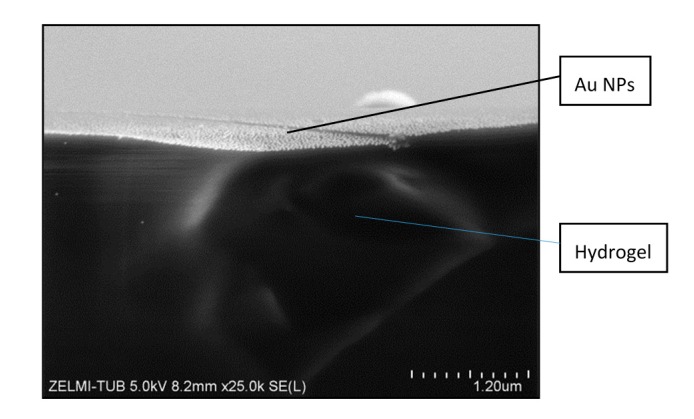
SEM of a cross-section view of Au NPs after transfer onto the PEG-hydrogel surface.

After creating a pattern of Au NPs on the silicon wafer, it was then transferred onto the PEG-hydrogel. Hereby, as mentioned before, the PEGprepolymer was cured on the Au NP patterned silicon wafer and the resulted PEG-hydrogel was wetted by some drops of water. During the swelling of the hydrogel the Au NPs were transferred selectively from the surface of the silicon wafer to the hydrogel. In Figure 5, an SEM image of a cross-section of a PEG-hydrogel after transfer of the Au NPs is shown. The bottom dark part is the hydrogel and on the top part a monolayer of the distributed Au NPs on the surface of the hydrogel is observable.

**Figure 6:**
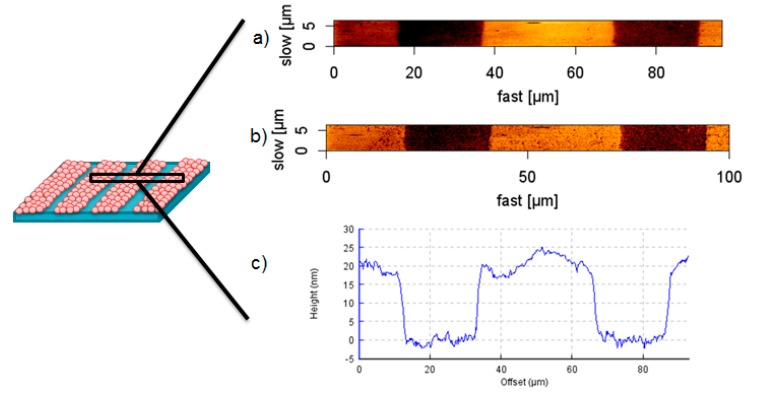
AFM (**a**) height image, (**b**) phase image and (**c**) cross-section profile of an Au NPs (20 nm) patterned PEG-hydrogel after transfer, pattern size (25 µm-25 µm-5 µm).

Height images of the patterned hydrogel after transfer of Au NPs in swollen state show a topography of 20 nm in lines of 25 µm width (Figure 6a,c), in correspondence with the used Au NPs and master sizes. Considering the phase image in Figure 6b, the surface apparently consists of two different materials with different surface-tip interactions, which underlines the successful pattern of the nanocomposite material containing Au NPs at the interface, and pure PEG as the base material.

**Figure 7:**
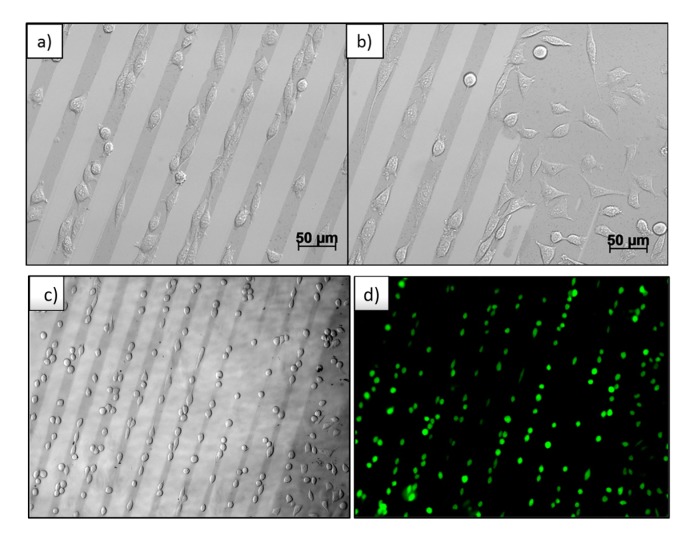
(**a**–**c**) Optical images of fibroblast adhesion on Au NPs patterned PEG-hydrogel. The darker lines correspond to the patterns of Au NPs; (**d**) live-dead assay.

Thus created micro patterns of Au NPs on PEG-hydrogels were cultured with mouse fibroblast cells L929 for 24 h. The result is shown in the optical microscopy images in Figure 7a–c. The micro-patterned Au NPs structures can be recognized as darker lines with respect to the pure PEG-hydrogel background. In Figure 7b, the edge of the Au NPs pattern is seen, where the Au NPs in previous processes of wet μ-CdP were partially detached from the silicon wafer and an area of undetached Au NPs had remained, which was then transferred to PEG. It is obviously seen that the cells selectively adhere on the Au NPs coated area. The cells are aligned and stretched with respect to the direction of the micro Au NPs lines (Figure 7a), whereas in the non-patterned regions the cells adsorbed and spread randomly on the Au NPs-coated area (Figure 7b).

The effect of cell adhesion on non-functionalized Au NPs was discovered in our previous publication [23] and is here corroborated by these results on the patterned Au NPs on PEG; specific bio-functionalization with cell adhesion molecules is not required for selective cell adhesion. Probably, cell adhesion-mediating molecules, e.g., proteins such as fibronectin and vinculin, bind (non-specifically) to the surface of the Au NPs, thereby replacing the citrate molecules. We have tried to detect such adsorption effects by using fluorescently labelled proteins, however, the proteins were found to diffuse into the gel, making the whole gel appearing fluorescent (data not shown). It is hypothesized that those proteins at the hydrogel surface are not useful for (integrin-mediated) cell adhesion, because they are not bound [26]. The stronger interaction of such proteins, and of many other possible components from the serum and cell culture medium with the Au NPs makes them better suitable as anchoring sites, hence enable focal adhesion contacts to be formed. In addition, the peculiar nano-size and high curvature of the Au NPs, combined with their nanoscale arrangement may further aid the formation of focal adhesions, with the cell membrane receptor complexes locally wrapping around the adhesive NPs [27].

In order to explore the effectiveness of using such small objects that are loosely adhering to the surface of the soft hydrogels to induce selective cell adhesion, we investigated if the same strategy would also be applicable employing Ag NPs instead of Au NPs. For that, we have synthesized citrate-stabilized Ag NPs with a comparable size (~30 nm) as the Au NPs (~20 nm), patterned them in the same way as the Au NPs, and studied the response of the fibroblasts. The particles were synthesized by the same seeded growth method as used for the Au NPs (see Supplementary Materials for details) and characterized by AFM and UV/Vis-spectroscopy (see Supplementary Materials, Figure S1). As expected, the wet µ-CdP process could be applied conveniently to make micro-patterns of Ag NPs on silicon first (see SEM images in Figure S2), which could then be transferred effectively to the PEG-hydrogel surface.

These micro-patterned PEG-gels were investigated in cell culture with L929 fibroblasts. The optical micrographs (Figure S3) demonstrate that cells were unable to adhere to the micro-lines of Ag NPs and were in fact dying. Control experiments further indicated cell death in the presence of Ag NPs in cell culture (Figure S4b). Both results underline that unlike Au NPs, Ag NPs are not cytocompatible. Further, systematic cytotoxicity studies should reveal whether this is generally the case for Ag NPs, with different sizes and shapes. Nanosilver is known to be antibacterial and can also invoke immunological responses in monocytes. These cellular responses are believed to be caused by Ag NPs interacting with cell membranes, damaging them and causing oxidative stress, i.e., the overproduction of reactive oxygen species (ROS) [28,29].

In Figure 7d, the live-dead assay is shown. Hereby, only the green fluorescent color is observed, corresponding to alive cells, and no red fluorescence—corresponding to dead cells—was detected. This indicates the great cell viability and underlines the cyto-compatibility of these nanocomposite biomaterials towards mouse fibroblasts L929 cells. In the Supplementary Materials more cytotoxicity results are discussed (Figure S5).

**Figure 8:**
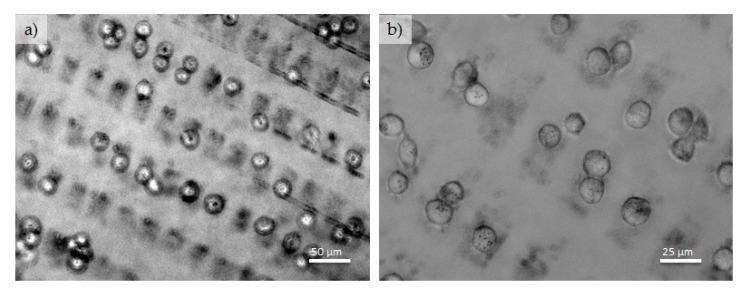
(**a**,**b**) Optical images of fibroblast adhesion on an Au NPs (120 nm) patterned PEG-hydrogel exhibiting rectangular patterns of Au NPs.

In Figure 8, rectangular patterned Au NPs on PEG-hydrogel with adhered fibroblasts are depicted. The sizes of the used silicon masters were; 25 µm-25 µm-5 µm and 20 µm-10 µm-5 µm. Again it is manifest that the fibroblasts preferentially adhere on the Au NPs-patterned areas, while the pure PEG areas are free of cells. On some rectangular areas of Au NPs, one or two cells are sitting next to each other, in a few cases also three cells are sitting in one rectangle structure (Figure 8). Moreover, the large, pure PEG area (25 µm lines and 10 µm line gaps) are virtually free of cells.

Finally, in order to broaden the scope of this versatile method to control selective cell adhesion to micro-patterns of Au NPs, we also investigated the cell adhesion of a different cell type, i.e., the osteoblast-like cells MT3T3E1. Figure S6 (Supplementary Materials) demonstrates that the osteoblast-like cells respond in a fashion similar to that observed for the fibroblasts: selective adhesion was observed only on the Au NPs-lines, while the pure PEG-background supported no cell adhesion. This implies that the wet micro-contact deprinting (wet µ-CdP) method can be applied in bone tissue engineering as well.

## 4. Conclusions

The Wet Micro-Contact Deprinting (wet μ-CdP) process is a fast, cheap, easy, and versatile process to get linear or rectangular micro-patterns of Au NPs on PEG-based hydrogels. For this process, desired micrometer-sized regions on Au NPs-coated silicon wafers were removed (deprinted). In that way, micro-patterns of Au NPs on silicon wafers were created. Afterwards, the Au NPs patterns were completely transferred from silicon wafers onto the surface of the hydrogel through swelling of the hydrogel in water without utilizing linker molecules. Though PEG-based materials are known to be resistant to protein or cell adsorption, on the thus created Au NPs micro-patterns mouse fibroblast cells L929 adhesions were investigated. It was observed that the cells selectively adhered on the Au NPs loaded areas and the pure PEG-hydrogel material was absolutely free of cells. Also, using this method, we managed to create square and rectangular Au NPs island patterns in micrometer ranges on a PEG background. In that way, immobilization of cells (from single cells to maximally three cells per area) with defined separations was achieved.

Not only cells but also biomolecules, such as proteins or enzymes, could be bound selectively to the nano-composite PEG surface. These materials have highly promising applications ranging from electronic devices to biological or biomedical applications, due to conductivity, low toxicity, and specific optical properties (surface plasmons) of Au NPs and the non-toxicity and non-fouling properties of the PEG-based materials. They are especially useful materials for protein, enzyme, or cell based biosensor applications. The single cell Raman spectroscopy, where the Au NPs pattern provides specific adhesions for biomolecules and enhances the Raman signals at the same time is another highly promising application.

## Supplementary Materials

The following are available online at www.mdpi.com/2073-4360/9/5/176/s1, Figure S1: AFM height images and cross section profiles and UV-Vis spectrum of citrate capped Ag NPs, Figure S2: (a–d) SEM images of the micro-patterned Ag NPs on SI-wafer using wet µ-CdP approach, Figure S3: Optical micrographs of L929 cell culture on Ag NPs micro-patterns, Figure S4: (a) L929 cell adhesion on TCPS as control; (b) L929 cells cultured in Ag NP solution on TCPS, Figure S5: Live/dead fluorescence images of L929 cells; (a) on TCPS; (b) on Au NPs patterned PEG-hydrogel; (c) in Au NPs solution and (d) statistical histogram of the viability of the cells, Figure S6: (a,b) MT3T3 E1 cell adhesion on Au NPs patterned PEG-hydrogel; sizes of the silicon Master: (10 µm-50 µm-10 µm). The cells are encircled for ease of recognition.
