# Supplementary material for: Novel Wet Micro-Contact Deprinting Method for Patterning Gold Nanoparticles on PEG-Hydrogels and Thereby Controlling Cell Adhesion
Source: Polymers (Basel). 2017 May 15;9(5):176. doi: 10.3390/polym9050176 (PMC6432305; doi:10.3390/polym9050176)
Supplement: Supplementary file 1 [file polymers-09-00176-s001.pdf]

Supplementary Materials: Novel Wet Micro-Contact Deprinting Method for Patterning Gold Nanoparticles on PEG-Hydrogels and Thereby Controlling Cell Adhesion

Cigdem Yesildag, Christoph Bartsch, Gonzalo de Vicente and Marga C. Lensen

1. Experimental Procedure for the Synthesis of Ag NPs

Citrate stabilized silver nanoparticles (Ag NPs) were synthesized according to the protocol of Bastus et al. First of all, a mixture of 5 mM sodium citrate and 0.25 mM tannic acid was prepared in a 100 mL aqueous solution and heated for 15 min under vigorous stirring. After boiling had commenced, 1 mL of 25 mM $AgNO_3$ was injected into this solution. The color of the solution turned bright yellow immediately. The achieved Ag NPs were purified by centrifugation at 8000 rpm for 20 min in order to remove tannic acid and the resultant Ag NPs were redispersed in Milli-Q-water. All the chemicals were purchased from Sigma Aldrich (Steinheim, Germany) and used without purification.

2. AFM Studies and UV/Vis-Spectroscopy of Ag NPs

Besides the originally designed Au NPs micro-patterned PEG-hydrogels, we investigated fibroblast L929 cell adhesion on micro-patterns of silver nanoparticles (Ag NPs) on PEG as well.

For that, we synthesized around ~20 nm-sized citrate-capped Ag NPs using the Bastus Ag NPs synthesis method (*vide supra*). These Ag NPs were micro-patterned first on Si-wafer and then transferred onto the surface of PEG-hydrogels using the novel wet µ-CdP procedure. The synthesized Ag NPs were characterized by UV-Vis spectroscopy and AFM analysis (Figure S1).

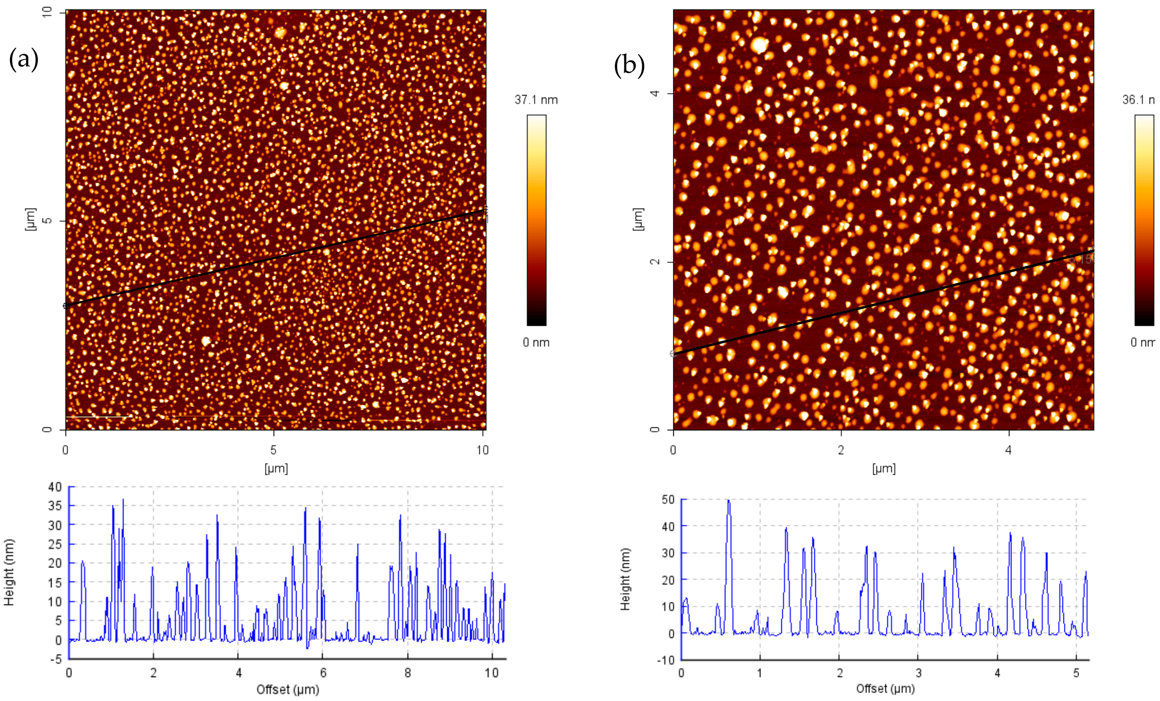

Figure S1. *Cont.*

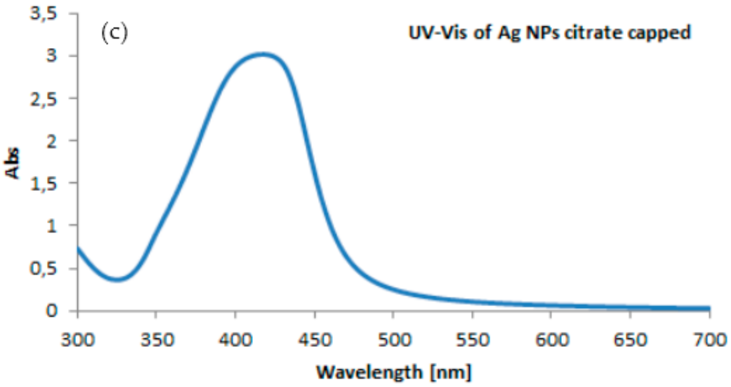

Figure S1. (**a**,**b**) AFM height images and cross section profiles and (**c**) UV-Vis spectrum of citrate capped Ag NPs.

In Figure a) and b) AFM analysis and c) UV-Vis spectrum of the synthesized Ag NPs are shown. From the cross section profiles it can be seen that the heights of the particles vary between around 10 and 40 nm and the UV-Vis spectrum has a maximum absorption at around 413 nm. Spherical Ag NPs with sizes of 28.0 ± 2.3 nm have an absorption peak of ~411.5 nm and Ag NPs with 31.6 ± 2.8 nm sizes have an absorption peak of ~418.5 nm. Considering these specifications the sizes of the synthesized particles are predicted to be between 28 and 32 nm.

3. SEM Characterization of Ag NPs Micro-Patterns on Silicon

The pattern was characterized by scanning electron microscopy (SEM):

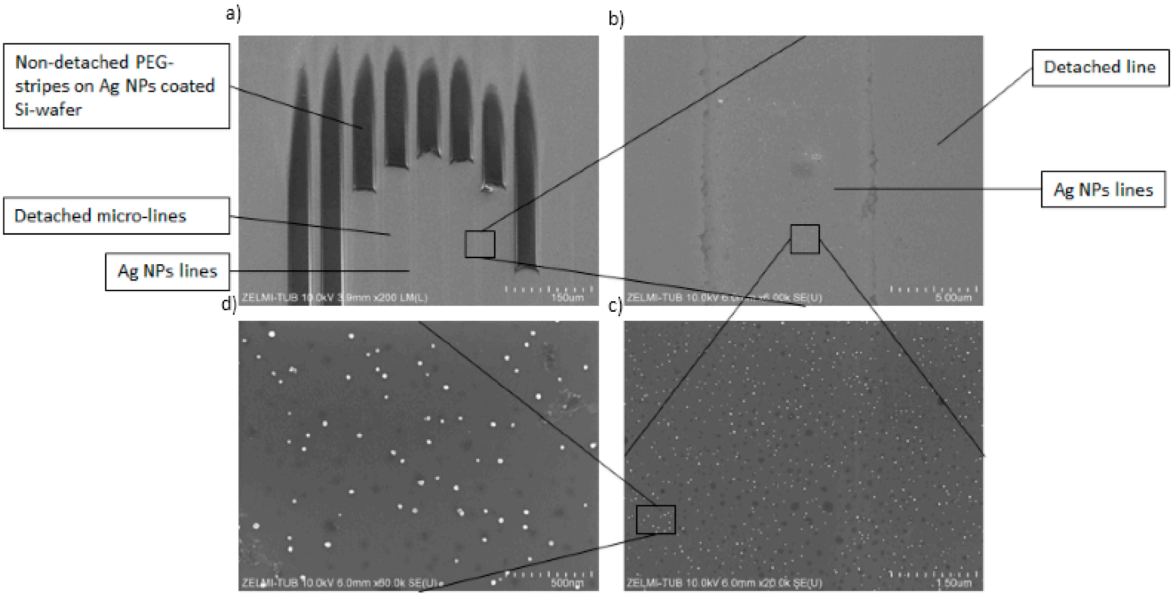

Figure S2. (**a**–**d**) SEM images of the micro-patterned Ag NPs on SI-wafer using wet µ-CdP approach.

In Figure S, SEM images of the micro-patterned Ag NPs on Si-wafer are shown. Hereby a master with pattern sizes of [10 µm-50 µm-10 µm] was used. In Figure Sa, the micro-lines of the Ag NPs on the Si-wafer can be recognized. The pattern sizes of the Ag NPs lines are comparable to the used master and have 10 µm widths and 50 µm distances. Furthermore, in Figure Sa, some non-detached PEG micro-stripes are visible. These are possibly not wetted PEG-stripes, which couldn´t be detached by swelling and stayed on the Si-wafer. In Figure Sb, an Ag NPs-line in an enlarged view is seen. The Ag NPs are only visible on this line and the surrounding area is free of Ag NPs, which indicates the applicability of the wet µ-CdP procedure also for Ag NPs patterning procedures. In Figure Sc, d, the distribution of the Ag NPs on the micro-lines are shown.

4. Optical Micrographs of Ag NPs Micro-Patterns of PEG-Hydrogel in Cell Culture with Fibroblasts

On these Ag NPs micro-patterned PEG-hydrogels, cell culture with L929 was investigated. Figure S3 depicts the optical micrographs, demonstrating the absence of cell adhesion:

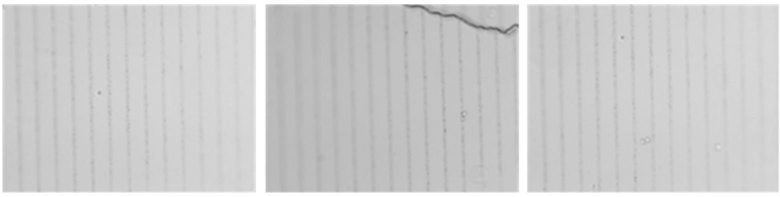

Figure S1. Optical micrographs of L929 cell culture on Ag NPs micro-patterns.

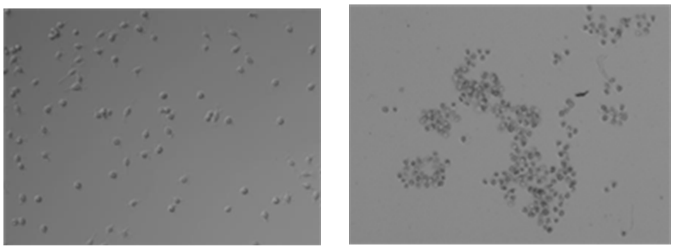

(**a**) (**b**)

Figure S2. (**a**) L929 cell adhesion on TCPS as control; (**b**) L929 cells cultured in Ag NP solution on TCPS.

As control experiments, cell culture was done on TCPS and in the as-prepared Ag NPs solution, which results are seen in Figure S2a, b. In Figure S2a, the cells are distributed, adhered and stretched on the TCPS surface, whereas in Figure S2b, the cells are round shaped and are predominantly aggregated, which indicates cell death. It can be preliminarily concluded that the prepared Ag NPs are cyto-toxic towards L929 fibroblast cells.

5. Live/Dead Staining Results on Control Samples (TCPS, Au NP-Suspensions) and Statistical Analysis

Cytotoxicity tests, e.g. live/dead staining were performed on TCPS as control experiment and also in Au NPs in solution and repeated several times. In Figure S5 further examples of the performed life/dead staining fluorescence images are shown; Figure S5a is performed on TCPS surface; Figure S5b on Au NPs micro-pattern on PEG-hydrogel and Figure S5c in Au NPs solution. In those images also a few red fluorescent cells (dead cells) are visible; those cells are usually always present, because of the normal cell cycle. In each case, the cell viability was above 99%; the statistics are shown in Figure S5d.

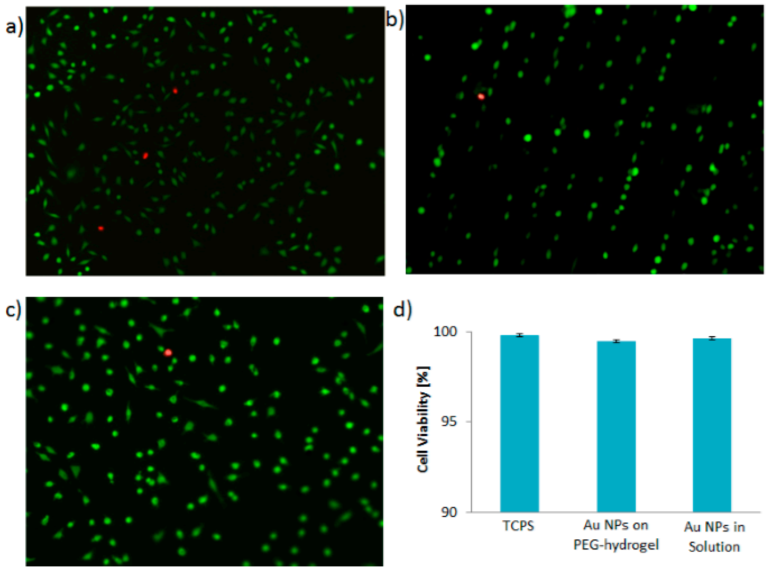

Figure S5. Live/dead fluorescence images of L929 cells; (**a**) on TCPS; (**b**) on Au NPs patterned PEG-hydrogel; (**c**) in Au NPs solution and (**d**) statistical histogram of the viability of the cells.

6. Cell Culture Studies of Osteoblast-Like Cells (MT3T3 E1) on Au NPs Micro-Patterned Hydrogels

On the originally designed patterned PEG-hydrogels exhibiting micro-lines of Au NPs, osteoblast-like cells (MT3T3 E1) were cultured. Comparable to L929 fibroblasts, also these cells display selective adhesion to the stripes of Au NPs, see Figure S6:

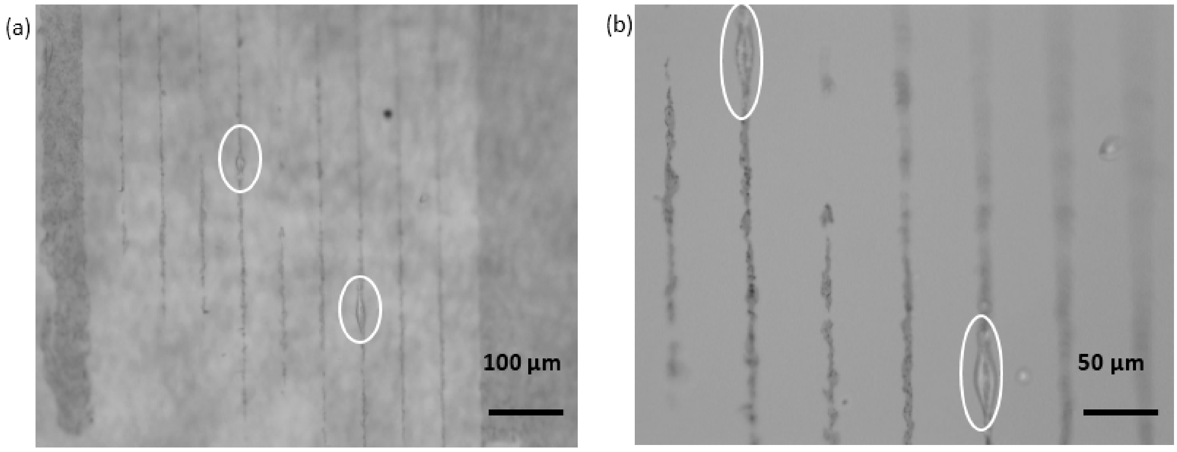

Figure S6. (**a, b**) MT3T3 E1 cell adhesion on Au NPs patterned PEG-hydrogel; sizes of the silicon Master: (10µm-50 µm-10 µm). The cells are encircled for ease of recognition.
